# *In Situ* Antibacterial Activity of Essential Oils with and without Alcohol on Oral Biofilm: A Randomized Clinical Trial

**DOI:** 10.3389/fmicb.2017.02162

**Published:** 2017-11-23

**Authors:** Victor Quintas, Isabel Prada-López, María J. Carreira, David Suárez-Quintanilla, Carlos Balsa-Castro, Inmaculada Tomás

**Affiliations:** ^1^Oral Sciences Research Group, Department of Surgery and Medical Surgical Specialties, School of Medicine and Dentistry, Health Research Institute of Santiago (IDIS), Universidade de Santiago de Compostela, Santiago de Compostela, Spain; ^2^Centro Singular de Investigación en Tecnoloxías da Información, Health Research Institute of Santiago (IDIS), Universidade de Santiago de Compostela, Santiago de Compostela, Spain

**Keywords:** anti-infective agents, local, biofilm, dental plaque, essential oils, microscopy, fluorescence

## Abstract

Currently, there is little evidence on the *in situ* antibacterial activity of essential oils (EO) without alcohol. This study aimed to evaluate *in situ* the substantivity and antiplaque effect on the plaque-like biofilm (PL-biofilm) of two solutions, a traditional formulation that contains EO with alcohol (T-EO) and an alcohol-free formulation of EO (Af-EO). Eighteen healthy adults performed a single mouthwash of: T-EO, Af-EO, and sterile water (WATER) after wearing an individualized disk-holding splint for 2 days. The bacterial viability (BV) and thickness of the PL-biofilm were quantified at baseline, 30 s, and 1, 3, 5, and 7 h post-rinsing (Test 1). Subsequently, each volunteer wore the splint for 4 days, applying two daily mouthwashes of: T-EO, Af-EO, and WATER. The BV, thickness, and covering grade (CG) of the PL-biofilm were quantified (Test 2). Samples were analyzed by confocal laser scanning microscopy after staining with the LIVE/DEAD® BacLight™ solution. To conduct the computations of the BV automatically, a Matlab toolbox called Dentius Biofilm was developed. In test 1, both EO antiseptics had a similar antibacterial effect, reducing BV after a single rinse compared to the WATER, and keeping it below baseline levels up to 7 h post-rinse (*P* < 0.001). The mean thickness of the PL-biofilm after rinsing was not affected by any of the EO formulations and ranged from 18.58 to 20.19 μm. After 4 days, the T-EO and Af-EO solutions were significantly more effective than the WATER, reducing the BV, thickness, and CG of the PL-biofilm (*P* < 0.001). Although, both EO antiseptics presented a similar bactericidal activity, the Af-EO rinses led to more significant reductions in the thickness and CG of the PL-biofilm than the T-EO rinses (thickness = 7.90 vs. 9.92 μm, *P* = 0.012; CG = 33.36 vs. 46.61%, *P* = 0.001). In conclusion, both essential oils antiseptics had very high immediate antibacterial activity and substantivity *in situ* on the 2-day PL-biofilm after a single mouthwash. In the 4-day PL-biofilm, both essential oils formulations demonstrated a very good antiplaque effect *in situ*, although the alcohol-free formula performed better at reducing the biofilm thickness and covering grade.

**This Clinical Trial was registered at clinicaltrials.gov with the number NCT03146390 URL: https://clinicaltrials.gov/ct2/show/NCT03146390.**

## Introduction

The accumulation of bacterial biofilms on tooth surfaces results in two of the most prevalent infectious diseases—caries and periodontitis. Although prevention and control of these diseases can be achieved by the daily mechanical removal of biofilms, many people are either unable or unwilling to practice these procedures as regularly or as efficiently as necessary. There is, therefore, great interest in the possibility of using chemicals to replace or augment, mechanical preventive, and therapeutic procedures (Marsh and Bradshaw, 1993; ten Cate and Marsh, 1994; Newman, 1996).

The active ingredients present in the mouthwashes that are most commonly used in the oral cavity include: chlorhexidine, combinations of essential oils (EO), triclosan, cetylpyridinium chloride, and various metal salts such as zinc compounds and stannous fluoride. Of all of these, chlorhexidine mouthwashes are considered to be the gold standard, as they have thus far been the most effective in microbiological and clinical studies (McDonnell and Russell, 1999; Tomás et al., 2008; von Ohle et al., 2010). However, their well-known undesirable secondary effects, mainly after regular use (Van Strydonck et al., 2012), have led to the scientific community exploring the existence of effective alternatives, especially when continuous daily use is required. Accordingly, EO have been found to be as effective as chlorhexidine at controlling gingival inflammation after 6 months of use, although the latter performs better at reducing plaque levels (Van Leeuwen et al., 2011; Neely, 2012).

EO are composed of a wide variety of products. As a consequence, their antimicrobial activity is related to their composition, configuration, amount, and possible interactions (Lis-Balchin et al., 1998). The traditional formulation containing EO (T-EO) (Listerine® Mentol™, Johnson & Johnson)^1^ are a complex mix of phenolic compounds combined with various EO: 0.092% eucalyptol, 0.064% thymol, 0.06% methyl-salicylate, and 0.042% menthol. All of these are included in a hydroalcoholic vehicle containing between 21.6 and 26.9% alcohol (Fine, 1988). As a result, T-EO contains ethanol, which is a chemical compound used to dissolve and stabilize the numerous substances present in the rinse. The concentration of ethanol present in the T-EO rinses is more than 20%. Such amounts have been found to be sufficient to dissolve the EO, but insufficient when it comes to having a direct antibacterial effect (Sissons et al., 1996; Marchetti et al., 2009). In fact, the manufacturer presents the alcohol contained in the rinse (21.6%) as being, inter alia, an inactive ingredient in its formula. Over the years, the use of ethanol in mouthwashes, as well as their effects on the surfaces of composite restorations (Penugonda et al., 1994) and their possible role in the development of oropharyngeal cancer, have been discussed (Smigel, 1991; Llewelyn, 1994). A direct cause-and-effect correlation between the development of oropharyngeal carcinoma and the use of alcohol-containing rinses has not been demonstrated (Moazzez et al., 2011; Bagán et al., 2012; Gandini et al., 2012), and probably never will be (at least by epidemiological studies; Lachenmeier, 2012). However, it is considered desirable to eliminate ethanol from daily mouthwashes, especially for pediatric populations and patients at higher risk for oral cancer (McCullough and Farah, 2008; La Vecchia, 2009). Furthermore, the fact that the alcohol is present has meant that some clinical practitioners do not prescribe the T-EO due to this controversy (Vlachojannis et al., 2013). All of this has led to the development of a new alcohol-free formulation of EO (Af-EO) (Listerine® Zero™, Johnson & Johnson)^1^.

The composition of Af-EO is the same regarding their active ingredients (eucalyptol, thymol, methyl salicylate, and menthol), but sodium fluoride has been added to the mixture. Some differences are found in their inactive ingredients. These are based on the alcohol contained in the T-EO solution, which is not present in the Af-EO rinse, and the presence of propylene glycol, sodium lauryl sulfate and sucralose in the Af-EO solution, but not the T-EO rinse.

Two different concepts should be taken into account to measure the efficacy of antiseptics against dental plaque: the substantivity and the antiplaque effect. The substantivity of an oral antiseptic is defined as the prolonged adherence to oral surfaces (teeth and mucosa) and its slow release at effective doses which guarantee the continuation of the antimicrobial activity (Manau Navarro and Guasch Serra, 2003). The more substantivity an oral antiseptic has, the better. To study this *in situ*, the most popular models are those that analyze the effect that a single mouthwash has on a biofilm of more than 24 h (García-Caballero et al., 2013; Quintas et al., 2015b).

The second concept that should be studied, the antiplaque effect, is defined as the capacity that an agent has to prevent the formation of bacterial aggregates (plaque) on oral surfaces. To study this *in situ* effect, models start from a baseline sample with levels of plaque near to zero in order to assess the power of the antiseptic when it comes to reducing the formation of bacterial plaque (normally dental plaque) compared to the control. A 6-month clinical study using a determinate antiplaque agent is necessary to tag an antiseptic as effective (Council on Dental Therapeutics, 1986). However, in the literature, there is an established model of 4 days of plaque regrowth with which authors can assess the inhibitory activity that different agents have; furthermore, this determines the relative efficacy of the different formulations as being predictive of the antiplaque effect of an antiseptic (Singh et al., 2013; Quintas et al., 2015a).

In addition, another important methodological aspect in the *in situ* study of an oral antiseptic is the need to conserve the oral biofilm intact at all stages, namely the formation, collection and analysis of the oral samples. The goal is to not interfere with the delicate three-dimensional structure of the oral biofilm, which has been proved to be essential in terms of the resistance to the effects of an antimicrobial agent (Wood et al., 2000; Beyth et al., 2010). For these reasons, the use of intraoral disks held in specially designed apparatus for biofilm formation combined with the application of confocal laser scanning microscopy has proved to be extremely valuable when it comes to analyzing the oral biofilm in its intact, hydrated natural state (Dong et al., 2010; Gosau et al., 2010; Gu et al., 2012; García-Caballero et al., 2013; Quintas et al., 2015a,b).

As Af-EO solution has come to the market, it seems appropriate to compare their antibacterial effects to those of traditional mouthwashes. Although, there are some studies evaluating these effects of T-EO and Af-EO (Marchetti et al., 2009, 2011, 2017a,b; Charles et al., 2012; Pizzo et al., 2013; Ulkur et al., 2013), none of them have assessed and compared their substantivity and antiplaque impact in an *in situ* model of a non-destructured oral biofilm (PL-biofilm). For this reason, the aim of the present study was to compare the *in situ* antibacterial activity (immediate effect, substantivity and antiplaque effect) of EO with and without alcohol on the PL-biofilm.

## Materials and methods

This research is a randomized, double-blind, crossover study of the antibacterial and antiplaque efficacy of two available EO solutions: a traditional formula of EO with alcohol (T-EO) and an alcohol-free formula of EO (Af-EO). The supporting CONSORT checklist is available as supporting information (Supplementary Table 1). The study received the approval of the Clinical Research Ethics Committee of Galicia (number 2014/008) and was registered at clinicaltrials.gov with the number NCT03146390. URL: https://clinicaltrials.gov/ct2/show/NCT03146390.

The “*a priori*” sample size calculation was performed using the program G^*^Power 3.1.5 (Faul et al., 2007). The following statistical criteria were established: (1) an effect size of 0.7; (2) an alpha error of 0.05; and (3) a statistical power of 80%. A sample size of 19 subjects was required by these criteria and the application of the Wilcoxon signed-rank test to analyze the differences in the microscopic parameters between two rinsing protocols.

**Figure 1:**
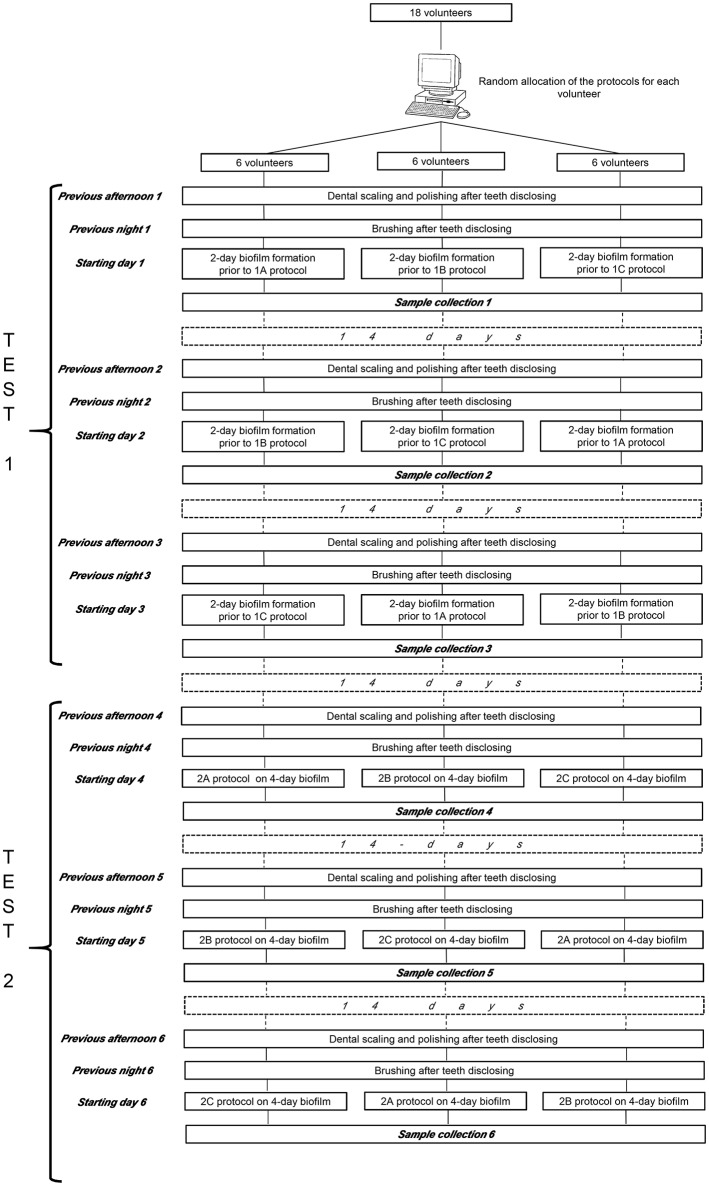
Protocol of the study.

The participants were selected among dental students at the School of Medicine and Dentistry of Santiago de Compostela (Universidade de Santiago de Compostela, Spain), where volunteer enrollment was sought by inviting responses to advertisements displayed in the faculty hall asking for participation in a research study. All these volunteers were assessed by the same trained clinician to ensure that they fulfilled all the inclusion and exclusion criteria that were applied in our group's previous publications (García-Caballero et al., 2013; Prada-López et al., 2015a,b; Quintas et al., 2015a,b). The inclusion criteria were the following: systemically healthy adult volunteers aged between 20 and 45 with a good oral health status, namely a minimum of 24 permanent teeth with no evidence of gingivitis or periodontitis (Community Periodontal Index score = 0; World Health Organization, 2013) and an absence of untreated caries at the start of the study. The following exclusion criteria were applied: smoker or former smoker, the presence of dental prostheses or orthodontic devices, antibiotic treatment, or routine use of oral antiseptics in the previous 3 months, and the presence of any systemic disease that could alter the production or composition of saliva. Before the start of each test or experiment, a full mouth scaling with ultrasonic instruments and teeth polishing with a rubber cup after dental disclosure were performed by the same trained clinician on all the selected participants (Figure 1). Written informed consent was obtained from all the volunteers. To achieve the aims of the study, all the participants performed two different tests.

To test the antibacterial activity of the two EO solutions, an *in situ* model of PL-biofilm growth was used. An individualized thermoplastic splint called intraoral disk-holding splint (IDODS) (García-Caballero et al., 2013; Prada-López et al., 2013, 2015a,b,c, 2016; Quintas et al., 2015a,b) with a capacity to hold a total of six glass disks was made for each of the volunteers.

### Test 1 (substantivity)

The first experiment consisted of evaluating the immediate antibacterial effect and substantivity of the T-EO and Af-EO solutions. The volunteers wore an IDODS for 48 h to enable growth of the PL-biofilm. They were allowed to remove it from the oral cavity only during meals and to perform oral hygiene measures (when it had to be stored in a provided opaque container in humid conditions). In order to not disturb the formation of the PL-biofilm, the volunteers could not use any toothpaste or mouthwash as a complement to the mechanical removal of bacterial plaque.

After 48 h, the glass disks were withdrawn one by one from the splint from each volunteer (from right to left in a distal-mesial direction) at baseline, 30 s, and 1, 3, 5, 7 h after performing the different mouthwashes. These mouthwashes were practiced under the investigator's supervision with the IDODS present in the oral cavity, and they were:

1A) A single, 30-s mouthwash with 20 mL of sterile water (negative control) (M-WATER).
-OR
1B) A single, 30-s mouthwash with 20 mL of a traditional EO formulation (Listerine® Mentol™, Listerine®, Johnson & Johnson, Madrid, Spain) (M-T-EO).
-OR
1C) A single, 30-s mouthwash with 20 mL of an alcohol-free EO solution (Listerine® ZERO™, Listerine®, Johnson & Johnson, Madrid, Spain) (M-Af-EO).

On the day of the experiment, the volunteers were not allowed to eat or drink during the tests. Collection of the different PL-biofilm samples started at 11:50 a.m. (baseline sample) and finished at 7:00 p.m. (the final sample was obtained 7 h after using the mouthwash).

Using an internet-based, balanced randomization system (Dallal)^2^, which indicated the mouthwash that each subject should use first, second and third, all the volunteers used the three mouthwashes, with a rest period of 2 weeks between each application (Figure 1).

### Test 2 (antiplaque effect)

The second experiment consisted of evaluating the antiplaque effect of both EO formulas. During 96 h, each volunteer wore the splints with the glass disks, withdrawing them from their oral cavity only during meals (they were stored in an opaque container in humid conditions) and to perform oral hygiene procedures involving only the mechanical removal of bacterial plaque with water, without the use of any toothpaste or mouthwash.

Using the permitted mechanical oral hygiene measures (without the IDODS), the volunteers performed the different protocols based on the manufacturers' instructions (with the IDODS in the oral cavity) over 4 days in the morning (8.30) after breakfast and at night (22.00) after dinner. These protocols were:

2A) A 30-s mouthwash with 20 mL of sterile water (negative control) (4D-WATER).
-OR
2B) A 30-s mouthwash with 20 mL of a traditional EO formulation (Listerine® Mentol™, Listerine®, Johnson & Johnson, Madrid, Spain) (4D-T-EO).
-OR
2C) A 30-s mouthwash with 20 mL of an alcohol-free EO solution (Listerine® ZERO™, Listerine®, Johnson & Johnson, Madrid, Spain) (4D-Af-EO).

The collection of the samples was carried out individually at 8 a.m. in the morning so that those of each volunteer were analyzed on different days. It was determined that a minimum of 10 h should have elapsed since the last use of the mouthwash the previous night.

In this test, mouthwashes carried out by the volunteers were not supervised, but they were instructed to use a measured volume of the allocated solution. To assess the subject's compliance with the rinsing protocol, the bottles containing the rinse were weighed before they were given to the volunteers. After the 4-day period, they were asked to bring the bottles back with the remaining mouthwash, and these were weighed again.

Using an internet-based, balanced randomization system (Dallal), which indicated the rising protocol that each subject should use first, second and third, all the volunteers used the three regimes, with a rest period of 2 weeks between each protocol (Figure 1).

### Processing of the samples of the PL-biofilm

As the glass disks were removed from the splint, they were immediately immersed in 100 μL of a fluorescence solution of LIVE/DEAD® BacLight™ and kept in a dark chamber at room temperature for 15 min. Microscope observations were performed by a single investigator who was unaware of the study design using a Leica TCS SP2 laser scanning spectral confocal microscope (Leica Microsystems Heidelberg GmbH, Mannheim, Germany) with an HCX APOL 63x/0.9 water-immersion lens.

Four fields considered to be representative of the entirety of the samples were selected by an observer who was blind to the study's conditions. Fluorescence emission was determined in a series of XY images in which each image corresponded to each of the Z positions (depth). The optical sections were scanned in 1 μm sections from the surface of the biofilm to its base, measuring the maximum thickness of the field and subsequently the mean thickness of the biofilm of the corresponding sample. The maximum biofilm thickness of each field was divided into three zones or equivalent layers: the outer layer (layer 1), the middle layer (layer 2), and the inner layer (layer 3).

The capture of the data was done with the same settings in all cases. The spatial scan mode (XYZ) and the 1,024 × 1,024 pixels scan format resolution were used. The Argon-ion and DPSS laser were used at a 13 and 78% of maximum intensity, respectively. The values for the pinhole, zoom and scan speed were 121.58 microns, 1 and 400 Hz, respectively. The only values that were different depending on the sample were the offset (range between −1 and 1%) and PMT gain which was different for channel red and green, being in general terms, higher for green than for red (test and positive control), due to the fact that there was more presence of red than green signal, being for the negative control the opposite. These values were always adjusted to get a good quality capture without background noise, avoiding excessive saturation of the brightest pixels of the image. As the technician was blinded to the experiment, they were advised to make the adjustments always consistent with what was seeing by the objective of the microscope, obtaining an image which was the closest as possible to reality.

The quantification of bacterial viability (BV) in the series of XY images was determined using a cytofluorographic analysis (Leica confocal software). In this analysis, the images of each fluorochrome were defined as “channels” (SYTO 9 occupies the green channel and Propidium Iodide the red channel). To conduct the computations automatically, a Matlab toolbox called Dentius Biofilm was developed. The main program reads all the images from an experiment, organized in a folder tree, with the image folder at the top, the experiment folder below this and all the patient folders at the bottom. The program automatically computes the number of disks, fields and 1 μm sections from the images stored in each patient's folder.

The program considers the parameters fixed by experts: the BV is characterized by a high value in the green channel (over 100, with a range between 0 and 255) and a low value in the red channel (below 100). Bacteria are considered not to be viable if the values are high in the red channel (over 100) and low in the green channel (below 100). Values that are high in both channels (over 100) are visually orange and are considered non-viable bacteria. The program counts the number of pixels under these conditions to compute the BV percentage for each 1 μm section (viable bacteria/viable bacteria + non-viable bacteria × 100). Determination of the mean BV percentage in each field required sections with a minimum biofilm area of 250 μm^2^ (~4,750 pixels).

The program also considers the case where epithelial nuclei are present. These are characterized by compact red areas with a size that is greater than the bacteria, and these red points must not be counted as non-viable bacterial population. To eliminate these pixels, the program disregards epithelial cells, which are characterized by: having a high value in the red channel, an area >200 pixels, compact regions with a solidity >0.7, and a minimum value of the mean intensity of 180. These parameters were fixed using a training set. With this methodology, there could be some misdetections, but the effect on the BV was very low, as what was important was the elimination of large areas with a high intensity.

All the results obtained for each section, field and disk from each patient were stored in a worksheet to be analyzed by the researchers. The BV percentage was also stored before and after eliminating the epithelial nuclei and their properties to localize them over the image. The mean BV percentage of the biofilm was calculated for the corresponding sample and for each biofilm layer.

In Test 2, apart from the thickness and BV, the covering grade (CG) was also assessed. This parameter is the percentage of the surface substrate covered by the biofilm. The cytofluorogram itself was used for this purpose. From the maximum projection (superposition of all captured planes) of each of the analyzed fields, the CG percentage was obtained by calculating the sum of the bacterial mass (viable and non-viable) with regard to the total surface of the field (% positive within the total area).

### Statistical analysis

The statistical analyses were performed using the R software (R Core Team, 2016). The Shapiro–Wilk test was performed to analyze the distribution of the quantitative variables associated with the PL-biofilm (thickness, BV and CG), showing mostly these microscopic parameters a non-normal distribution in both tests.

In Test 1 (substantivity) and Test 2 (antiplaque effect), the Friedman test was used for intra-mouthwash and inter-mouthwash comparisons using all the PL-biofilm samples (including differentiating between the three biofilm layers). In both tests, the Wilcoxon signed-rank test was used for pairwise comparisons (with Bonferroni adjustment) of the intra- and inter-mouthwash results (including differentiating between the three biofilm layers). The significance level established was a *P* < 0.05. In the Test 1, the Bonferroni-corrected *P-*values applied were <0.003 and <0.016, and in the Test 2, this value was <0.016.

## Results

A total of 30 volunteers were evaluated to obtain the calculated sample size (*n* = 19). When this number of participants meeting the inclusion and exclusion criteria was achieved, the enrollment process was ended. A total of 11 subjects were ineligible as they did not meet all of the inclusion criteria. All the participants performed both of the tests, although a subject was excluded after performing the Test 1 for an unexpected event (Supplementary Table 2, CONSORT flow diagram). No adverse effects were reported by them at any stage of the experiment. Eighteen subjects completed the rinsing protocols satisfactorily in both tests. In Test 2, the returns of each product suggested good compliance with the instructions.

### Test 1 (substantivity)

#### Influence of a single mouthwash of T-EO and Af-EO on the thickness of the PL-biofilm

Neither the T-EO nor the Af-EO antiseptics could reduce the thickness of the PL-biofilm of 48 h after a single application. Their baseline thicknesses were 21.81 ± 5.28 μm and 20.71 ± 4.13 μm, respectively. After a single mouthwash, the thicknesses were slightly reduced (20.19 ± 3.62 μm and 18.58 ± 3.14 μm, respectively), but did not achieve statistical significance.

#### Influence of a single mouthwash of T-EO and Af-EO on the bacterial viability of the PL-biofilm

**Figure 2:**
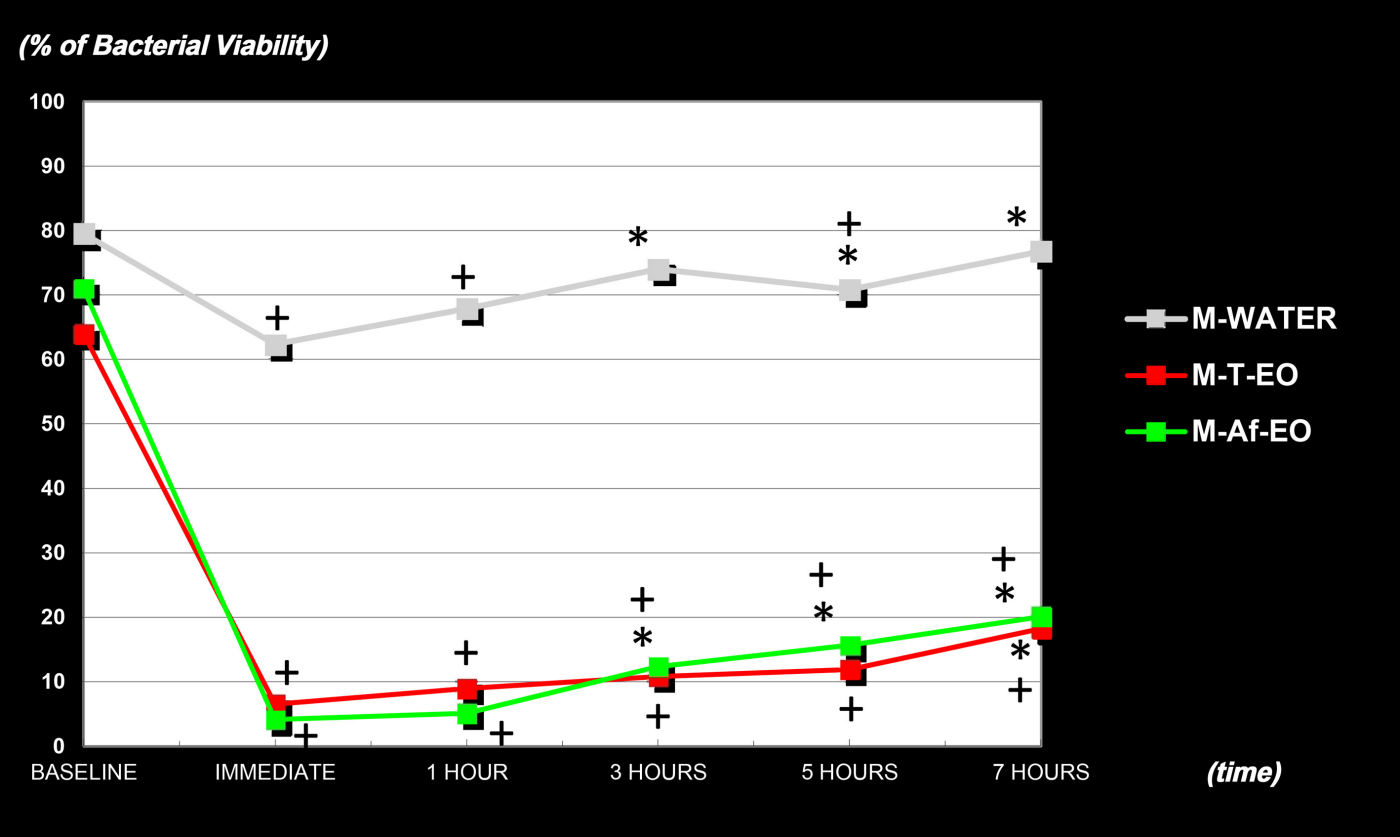
Percentages of bacterial viability of the PL-biofilm in baseline conditions, at 30 s, 1, 3, 5, and 7 h after a single mouthwash with sterile water (M-WATER), with essential oils with alcohol (M-T-EO), and with essential oils without alcohol (M-Af-EO). ^*^Statistically significant differences in regard to the 30-s sample (*P* < 0.003). ^+^Statistically significant differences in regard to the baseline (*P* < 0.003).

**Figure 3:**
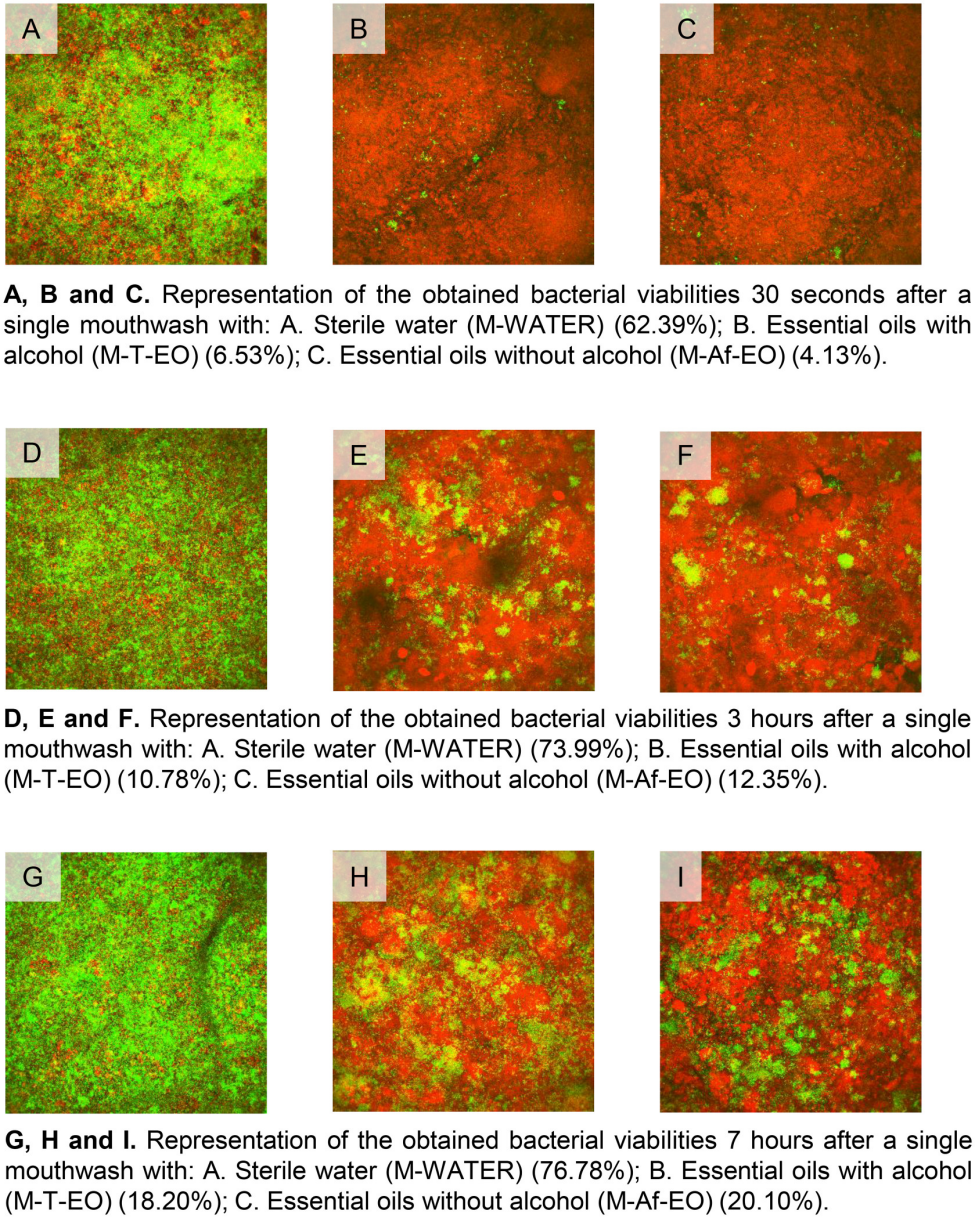
Representative images of the obtained bacterial viabilities at 30 s, 3 and 7 h after a single mouthwash with sterile water (M-WATER), traditional essential oils (M-T-EO), and alcohol-free essential oils (M-Af-EO).

The mean BV at baseline ranged between 63.99 ± 19.7% and 79.54 ± 5.31% for all the three rising protocols, with no statistical differences between them. Both EO formulations achieved similar results at all the time points measured. In fact, no differences were found between them from the immediate sample (30 s) to the 7-h sample (Figure 2). The EO formulations were effective at reducing the BV after a single mouthwash with respect to their respective baseline levels (BV at 30 s for M-T-EO and M-Af-EO = 6.53 ± 7.60% and 4.13 ± 3.89%, respectively; *P* < 0.001). These results were statistically lower than those from the M-WATER (62.39 ± 8.17%; *P* < 0.001). Both solutions were able to keep the BV under baseline levels for 7 h (BV at 7 h for M-T-EO and M-Af-EO = 18.20 ± 9.38% and 20.10 ± 10.27%, respectively; *P* < 0.001). Again, these findings were statistically lower than those from the M-WATER (BV at 7 h = 76.78 ± 4.40%; *P* < 0.001; Figures 2, 3).

In terms of BV recovery concerning the 30-s sample, significant recovery was not achieved until 7 h after the use of an M-T-EO rinse (BV at 30 s vs. at 7 h for M-EO = 6.53 ± 7.60% vs. 18.20 ± 9.38%; *P* < 0.001). However, for the M-Af-EO solution, significant recovery was identified in the 3-h sample (BV at 30 s vs. at 3 h for M-Af-EO = 4.13 ± 3.89% vs. 12.35 ± 8.86%; *P* < 0.001; Figure 2).

When it comes to differentiating between the three biofilm layers, the two EO antiseptics had lower BV levels in all the layers (Table 1). No significant differences were found in BV for the same biofilm layer between the EO formulations, with the outer layers being generally more viable than the inner ones in all the samples. There were no significant differences between the three layers for the M-T-EO rinse (BV at 30 s by layers = 6.67 ± 6.80% vs. 5.63 ± 8.15% vs. 7.29 ± 9.17%, respectively; *P* > 0.016) or between the deeper layers for the M-Af-EO rinse (BV at 30 s by layers 2 and 3 = 3.22 ± 3.24% vs. 3.51 ± 4.94%, respectively; *P* > 0.016; Table 1).

**Table 1:** Bacterial viability in 2-day PL-biofilm under basal conditions and in the samples collected at 30 s and 1, 3, 5, and 7 h after a single mouthrinse with: sterile water, traditional essential oils solution, and alcohol-free essential oils solution.

	**BASAL**	**30 S**	**1 H**	**3 H**	**5 H**	**7 H**
**BACTERIAL VIABILITY (%) OF THE 2-DAY PL-BIOFILM; MEAN ± STANDARD DEVIATION, MEDIAN (INTERQUARTILE RANGE)**						
**M-WATER**						
Layer 1 (outer layer)	86.82 ± 3.57 87.51 (2.18)	81.86 ± 6.63 80.48 (8.15)	85.46 ± 5.78 83.31 (6.89)	89.11 ± 6.13 91.90 (8.74)	86.26 ± 3.76 87.36 (6.29)	90.75 ± 3.45 89.54 (4.58)
Layer 2 (middle layer)	82.06 ± 5.02 (80.48 (3.87)	69.21 ± 7.83 68.53 (8.15)	73.94 ± 9.79 75.54 (6.02)	82.75 ± 5.98 80.91 (8.06)	77.20 ± 8.07 78.34 (11.84)	83.07 ± 4.25 84.73 (5.48)
Layer 3 (inner layer)	69.74 ± 16.21 73.69 (22.91)	36.08 ± 20.97 31.09 (26.52)	44.18 ± 14.80 44.34 (30.87)	50.12 ± 13.22 56.27 (15.80)	48.95 ± 17.78 52.45 (32.48)	56.53 ± 12.59 60.34 (9.10)
**M-T-EO**						
Layer 1 (outer layer)	75.54 ± 17.28 75.53 (19.71)	6.67 ± 6.80 4.44 (6.06)	9.90 ± 17.56 4.16 (4.80)	18.49 ± 14.37 16.36 (20.45)	22.35 ± 14.73 15.56 (28.93)	35.42 ± 19.00 35.55 (31.05)
Layer 2 (middle layer)	67.86 ± 21.95 75.91 (32.06)	5.63 ± 8.15 2.77 (3.53)	7.97 ± 18.16 1.69 (4.65)	8.04 ± 10.79 3.68 (7.61)	8.58 ± 7.78 5.45 (6.14)	13.26 ± 9.09 9.91 (11.96)
Layer 3 (inner layer)	48.56 ± 28.37 51.87 (32.67)	7.29 ± 9.17 1.72 (12.59)	9.02 ± 17.95 1.35 (6.06)	5.81 ± 9.26 0.52 (7.15)	4.76 ± 7.62 1.37 (4.29)	5.92 ± 9.21 2.56 (5.05)
**M-Af-EO**						
Layer 1 (outer layer)	84.74 ± 15.33 90.03 (9.74)	5.66 ± 5.72 3.88 (6.75)	10.01 ± 8.30 8.82 (10.24)	20.74 ± 14.73 18.56 (12.87)	27.69 ± 18.15 21.85 (21.27)	41.99 ± 18.96 46.53 (27.42)
Layer 2 (middle layer)	76.37 ± 19.38 81.93 (27.11)	3.22 ± 3.24 1.76 (5.33)	4.04 ± 4.25 2.46 (4.84)	9.70 ± 8.52 7.62 (10.00)	12.67 ± 10.22 8.32 (14.72)	14.28 ± 11.27 11.35 (14.96)
Layer 3 (inner layer)	51.95 ± 31.30 59.06 (56.86)	3.51 ± 4.94 2.01 (3.13)	1.23 ± 1.18 0.98 (1.30)	6.63 ± 8.39 4.23 (7.16)	6.53 ± 7.06 3.38 (8.52)	4.02 ± 6.50 1.50 (4.27)
**BACTERIAL VIABILITY, INTRA-MOUTHWASH ANALYSIS; STATISTICAL SIGNIFICANCE**						
**M-WATER**						
Layer 1 vs. Layer 2	*P* < 0.016	*P* < 0.016	*P* < 0.016	—	*P* < 0.016	*P* < 0.016
Layer 1 vs. Layer 3	*P* < 0.016	*P* < 0.016	*P* < 0.016	*P* < 0.016	*P* < 0.016	*P* < 0.016
Layer 2 vs. Layer 3	*P* < 0.016	*P* < 0.016	*P* < 0.016	*P* < 0.016	*P* < 0.016	*P* < 0.016
**M-T-EO**						
Layer 1 vs. Layer 2	*P* < 0.016	—	*P* < 0.016	*P* < 0.016	*P* < 0.016	*P* < 0.016
Layer 1 vs. Layer 3	*P* < 0.016	—	—	*P* < 0.016	*P* < 0.016	*P* < 0.016
Layer 2 vs. Layer 3	*P* < 0.016	—	—	*P* < 0.016	—	*P* < 0.016
**M-Af-EO**						
Layer 1 vs. Layer 2	*P* < 0.016	*P* < 0.016	*P* < 0.016	*P* < 0.016	*P* < 0.016	*P* < 0.016
Layer 1 vs. Layer 3	*P* < 0.016	—	*P* < 0.016	*P* < 0.016	*P* < 0.016	*P* < 0.016
Layer 2 vs. Layer 3	*P* < 0.016	—	*P* < 0.016	*P* < 0.016	*P* < 0.016	*P* < 0.016
**BACTERIAL VIABILITY, INTER-MOUTHWASH ANALYSIS; STATISTICAL SIGNIFICANCE**						
**M-WATER vs. M-T-EO**						
Layer 1 vs. Layer 1	*P* < 0.016	*P* < 0.016	*P* < 0.016	*P* < 0.016	*P* < 0.016	*P* < 0.016
Layer 2 vs. Layer 2	—	*P* < 0.016	*P* < 0.016	*P* < 0.016	*P* < 0.016	*P* < 0.016
Layer 3 vs. Layer 3	—	*P* < 0.016	*P* < 0.016	*P* < 0.016	*P* < 0.016	*P* < 0.016
**M-WATER vs. M-Af-EO**						
Layer 1 vs. Layer 1	*P* < 0.016	*P* < 0.016	*P* < 0.016	*P* < 0.016	*P* < 0.016	*P* < 0.016
Layer 2 vs. Layer 2	—	*P* < 0.016	*P* < 0.016	*P* < 0.016	*P* < 0.016	*P* < 0.016
Layer 3 vs. Layer 3	—	*P* < 0.016	*P* < 0.016	*P* < 0.016	*P* < 0.016	*P* < 0.016
**M-T-EO vs. M-Af-EO**						
Layer 1 vs. Layer 1	—	—	—	—	—	—
Layer 2 vs. Layer 2	—	—	—	—	—	—
Layer 3 vs. Layer 3	—	—	—	—	—	—

*Differences between the three biofilm layers, as well as intra-mouthrinse and inter-mouthrinse comparisons. M-WATER, A single, 30-s mouthwash with 20 mL of sterile water; M-T-EO, A single, 30-s mouthwash with 20 mL of a traditional essential oils solution; M-Af-EO, A single, 30-s mouthwash with 20 mL of an alcohol-free essential oils solution. —, no statistical significance*.

### Test 2 (antiplaque effect)

#### Influence of a 4-day protocol of T-EO and Af-EO mouthwashes on the thickness and covering grade of the PL-biofilm

**Figure 4:**
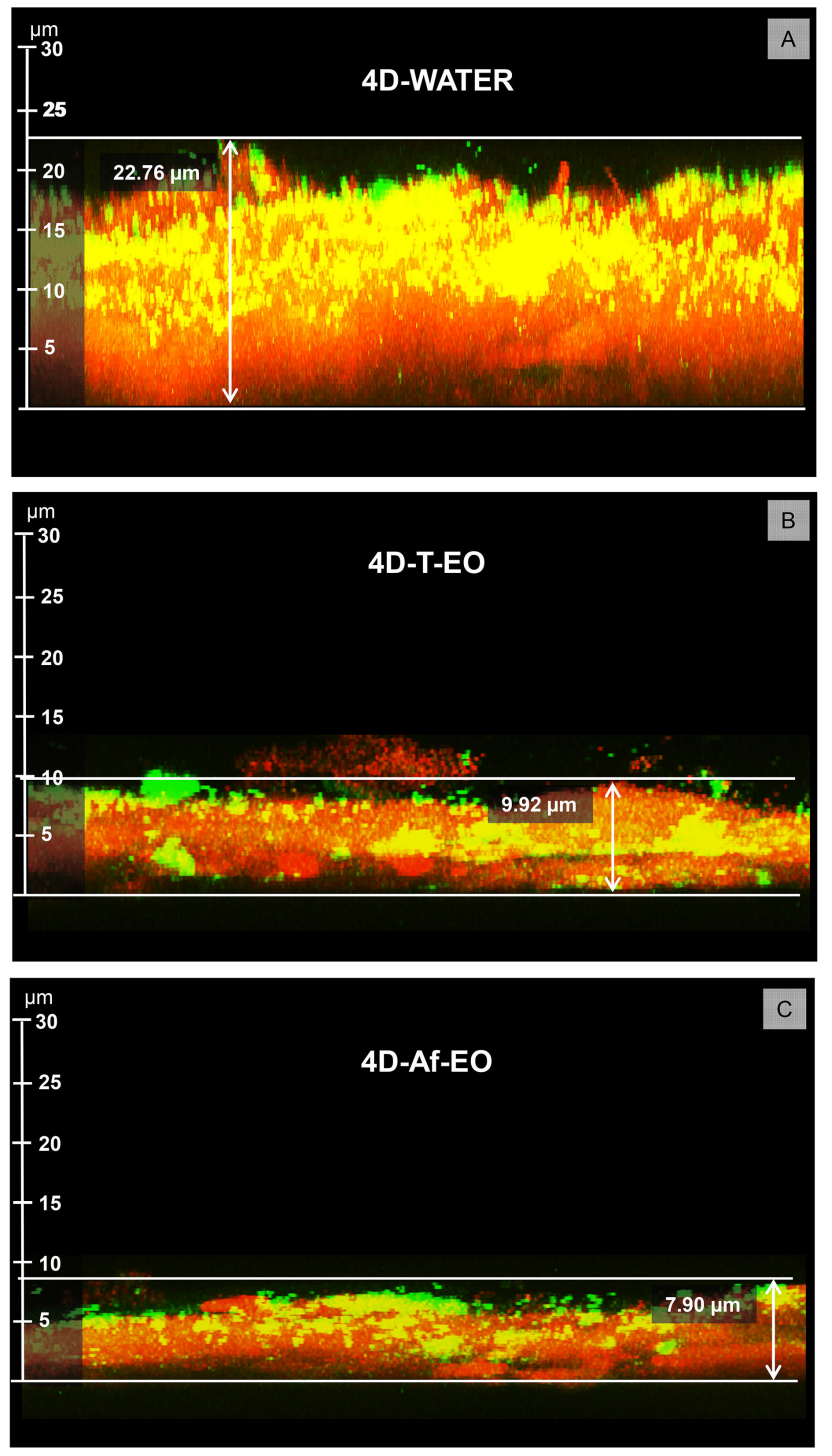
Representative images of the PL-biofilm thickness after 4 days of continuous use of: **(A)** Sterile water (4D-WATER), **(B)** Traditional essential oils (4D-T-EO), and **(C)** Alcohol-free essential oils (4D-Af-EO).

The Af-EO rinses were more effective than the T-EO formulation at reducing the thickness of the oral biofilm after 4 days of use (thickness for 4D-T-EO vs. 4D-Af-EO = 9.92 ± 2.87 μm vs. 7.90 ± 2.91 μm; *P* = 0.012), but both solutions were more powerful than the negative control (thickness for 4D-WATER = 22.76 ± 6.21 μm; *P* < 0.001; Table 2 and Figure 4).

**Table 2:** Bacterial viability, thickness, and covering grade of the PL-biofilm after 4 days of applying the three different rising protocols.

	**Bacterial viability (%)**	**Thickness (μm)**	**Covering grade (%)**
**BACTERIAL VIABILITY, THICKNESS, AND COVERING GRADE OF THE 4-DAY PL-BIOFILM MEAN ± STANDARD DEVIATION; MEDIAN (INTERQUARTILE RANGE)**			
4D-WATER	51.35 ± 5.38	22.76 ± 6.21	73.92 ± 17.49
	50.70 (6.69)	24.24 (5.72)	76.23 (15.59)
4D-T-EO	26.27 ± 14.61^*^	9.92 ± 2.87^*^	46.61 ± 19.12^*^
	22.68 (15.87)^*^	9.66 (3.80)^*^	45.49 (24.51)^*^
4D-Af-EO	31.08 ± 16.52^*^	7.90 ± 2.91^*^^§^	33.36 ± 12.01^*^^§^
	29.66 (14.61)	7.23 (2.29)^*^^§^	32.97 (14.92)^*^^§^

4D-WATER, Period of 4 days while the volunteer performs two daily mouthwashes with 20 mL of sterile water; 4D-T-EO, Period of 4 days while the volunteer performs two daily mouthwashes with 20 mL of a traditional essential oils solution; 4D-Af-EO, Period of 4 days while the volunteer performs two daily mouthwashes with 20 mL of an alcohol-free essential oils solution.

* Statistically significant differences in regard with the 4D-WATER, P < 0.016.

§ *Statistically significant differences in regard with the 4D-T-EO, P < 0.016*.

**Figure 5:**
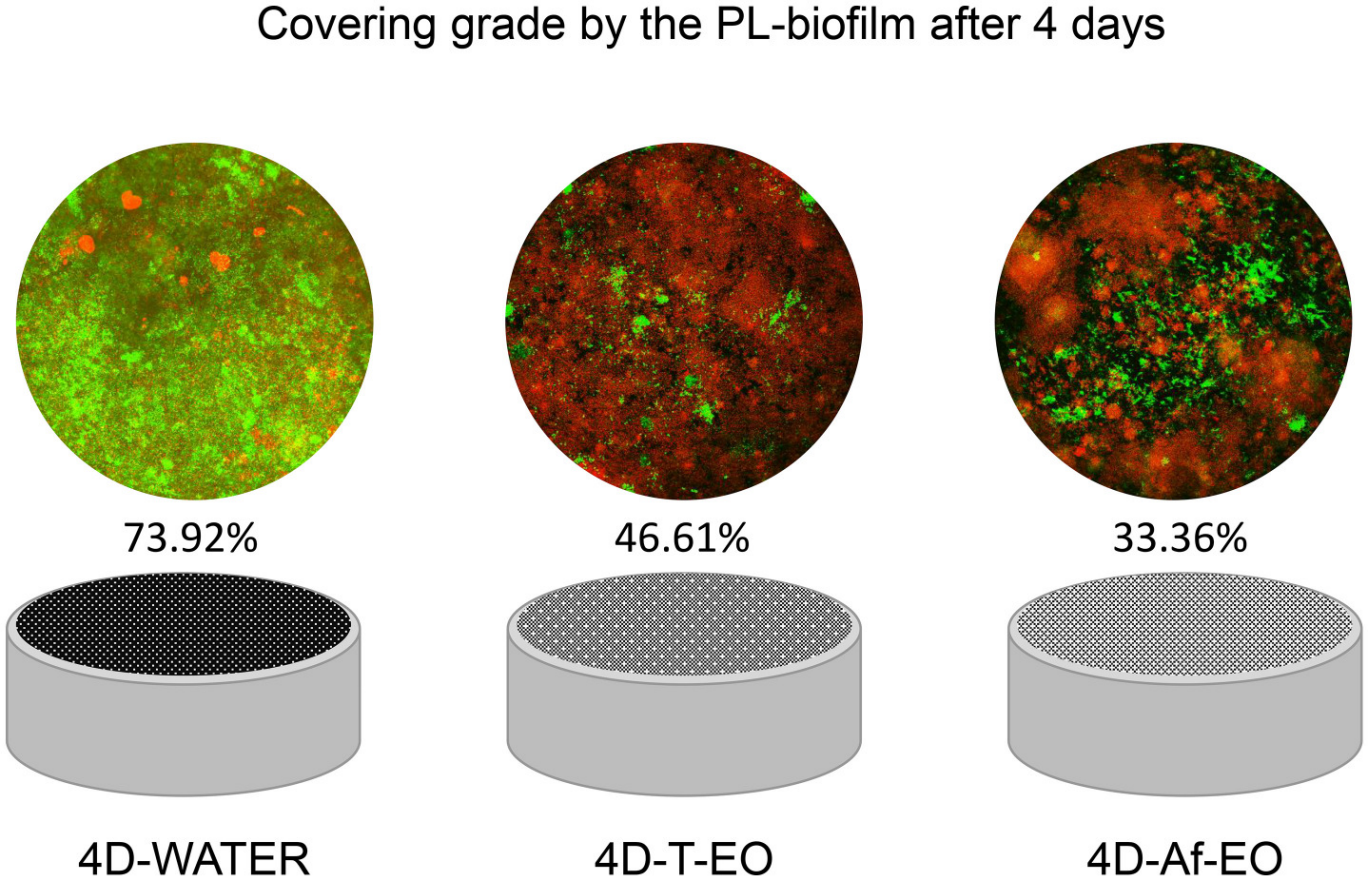
Representative images and graphics of the disks covering grade by the PL- biofilm after 4 days of continuous use of sterile water (4D-WATER), traditional essential oils (4D-T-EO), and alcohol-free essential oils (4D-Af-EO).

The Af-EO rinses were more effective than the T-EO solution at reducing the CG of the oral biofilm after 4 days of use (CG for 4D-T-EO vs. 4D-Af-EO = 46.61 ± 19.12% vs. 33.36 ± 12.01%, respectively; *P* = 0.001). The two EO solutions were significantly more effective than the negative control at reducing the CG (CG for 4D-WATER = 73.92 ± 17.49%; *P* < 0.001; Table 2 and Figure 5).

#### Influence of a 4-day protocol of T-EO and Af-EO mouthwashes on the bacterial viability of the PL-biofilm

The T-EO and Af-EO formulations after 4 days of use showed similar effectiveness in maintaining the BV at significantly lower levels than the negative control (BV for 4D-T-EO and 4D-Af-EO vs. 4D-WATER = 26.27 ± 14.61% and 31.08 ± 16.52% vs. 51.35 ± 5.38%, respectively; *P* < 0.001; Table 2). In terms of the BV by layers, the outer layers had significantly more BV than the inner ones in all the experiments. The T-EO and Af-EO formulations were significantly more effective at reducing the BV of layers 1 and 2 than the negative control, with layer 3 unaltered (BV for layer 1 = 40.10 ± 17.31% and 39.81 ± 19.09% vs. 82.47 ± 7.58%, respectively; BV for layer 2 = 24.32 ± 16.16% and 30.73 ± 17.06% vs. 51.76 ± 13.53%, respectively; *P* < 0.001 for all comparisons; Table 3).

**Table 3:** Bacterial viability of the PL-biofilm after 4 days of applying the three different rising protocols, differentiating by layers.

	**LAYER 1 (outer layer)**	**LAYER 2 (middle layer)**	**LAYER 3 (inner layer)**
**BACTERIAL VIABILITY (%) OF THE 4-DAY PL-BIOFILM BY LAYERS MEAN ± STANDARD DEVIATION; MEDIAN (INTERQUARTILE RANGE)**			
4D-WATER	82.47 ± 7.58^†^	51.76 ± 13.53^†^	19.83 ± 12.60^†^
	81.00 (11.38)^†^	45.30 (16.82)^†^	17.42 (8.19)^†^
4D-T-EO	40.10 ± 17.31^*^^†^	24.32 ± 16.16^*^^†^	14.40 ± 14.34^†^
	39.52 (21.20)^*^^†^	21.17 (15.98)^*^^†^	11.00 (17.00)^†^
4D-Af-EO	39.81 ± 19.09^*^^†^	30.73 ± 17.06^*^^†^	22.71 ± 17.05^†^
	35.14 (22.21)^*^^†^	29.26 (14.16)^*^^†^	17.37 (12.04)^†^

4D-WATER, Period of 4 days while the volunteer performs two daily mouthwashes with 20 mL of sterile water; 4D-T-EO, Period of 4 days while the volunteer performs two daily mouthwashes with 20 mL of a traditional essential oils solution; 4D-Af-EO, Period of 4 days while the volunteer performs two daily mouthwashes with 20 mL of an alcohol-free essential oils solution.

* *Statistically significant differences in regard with the 4D-WATER between the same layers (inter-mouthwash and intra-layer comparisons), P < 0.016*.

† *Statistically significant differences between the different layers of the same mouthwash (intra-mouthwash and inter-layer comparisons), P < 0.016*.

## Discussion

### Methodology approach

This is the first study to compare the antibacterial activity *in vivo* that T-EO and Af-EO solutions have on the non-destructured oral biofilm. From a methodological perspective, to perform all the BV computations automatically through a Matlab toolbox called Dentius Biofilm ensures that the experiment's findings are accurate, quick to obtain, reliable, and repeatable, which is very important when it comes to comparing results and extracting robust conclusions. In the substantivity study (test 1), mean BV data of each subject were obtained from analyzing ~160 sections of 1 μm (around 40 sections per field X four fields X one disk) for each evaluated moment (six-time moments: baseline, 30 s, 1, 3, 5, and 7 h). In the antiplaque study (test 2), when the negative control was applied, the mean BV values of each subject were obtained from evaluating ~1,200 sections (around 50 sections per field X four fields X six disks); when antiseptic solutions were used, the mean BV results of each subject were derived from the analysis of ~360 sections (around 15 sections per field X four fields X six disks).

In the present series, the BV was assessed by the Live/Dead® BacLight™ fluorescence assay. This solution stains the bacteria in red or green depending on the permeability of their membrane (propidium iodide which stains the cell in red only if the membrane permeability is altered). Given that the tested antiseptics act mostly at this cellular element, this viability method may be suitable for this type of study. Although, firstly conceived as a technique only valid for BV assessment of single species model (Invitrogen communication), its simplicity and good results also in *in situ* studies containing multiple bacterial species (Boulos et al., 1999; Ihalin et al., 2003; Tomás et al., 2009; Beyth et al., 2010; Dong et al., 2010; Gosau et al., 2010; García-Caballero et al., 2013; Tawakoli et al., 2013; Prada-López et al., 2015a,b; Quintas et al., 2015a,b) has produced the manufacturer to make a recommendation also for multiple species models (Live/Dead® BacLight™ User Manual)^3^.

Despite this, there has been some discussion about the reliability of this technique (Hannig et al., 2010; Tawakoli et al., 2013; Netuschil et al., 2014), mainly due to the fact that not only red and green bacteria appear in the analysis, but also orange regions appear with “unknown” interpretation (Berney et al., 2007). Hannig et al. (2010) considered that live/dead staining methods were reliable when evaluating antimicrobial agents activity. However, they continued to ask the question about “how dead is dead?” due to several stages of vitality which have been discussed and described in the literature (viable and culturable, viable but non-culturable, dormant, non-viable and pre-lytic, and avital dead bacteria). The exact differentiation of these stages is still one of the greatest challenges in modern microbiology (Decker, 2001).

Several attempts have been made to compare the Live/Dead® BacLight™ with the gold standard -traditional plaque cultures - (Boulos et al., 1999; Ihalin et al., 2003; Tomás et al., 2009; Tawakoli et al., 2013). One of these was performed several years ago by the authors (Tomás et al., 2009) in a study on the *in situ* substantivity of the chlorhexidine on the salivary bacteria. The mean BV obtained was compared with plaque cultures. A good correlation was observed between both techniques in the baseline and immediate samples. However, this correlation was lost as time passed, since plaque cultures could not detect the BV rise produced in the following samples. This led to an overestimation of the *in situ* effect of the antiseptic on cultivated bacteria, as previously demonstrated by other authors (Boulos et al., 1999; Ihalin et al., 2003). One of the reasons for this lack of correlation could be the fact that only 50% of the oral microbiota is culturable (Aas et al., 2005), which emphasizes the necessity of using viability assays (Tawakoli et al., 2013; Quintas et al., 2015b).

On the other hand, the BacLight™ system has been proposed as a reliable alternative when assessing BV in natural dental biofilm (Tawakoli et al., 2013). Furthermore, the fact of performing multiple measurements, as previously described, in every volunteer in each of the tests, being at the same time, a crossover study, considerably reduce the potential bias that could exist by the determination of the BV by this technique; in addition, it allows a better reproducibility in this type of studies.

Given that all microbiological techniques have their disadvantages, and although the presented results are coherent with the clinical reality, the authors recognize the convenience of contrasting and complementing the data obtained with BacLight™ fluorescence solution with other molecular or bacteriological techniques or even with other fluorescent dyes.

### Influence of a single mouthwash of T-EO and Af-EO on the thickness of the PL-biofilm

In the literature, very variable thickness measures have been identified in a non-disturbed 48 h-biofilm (Netuschil et al., 1998; Zaura-Arite et al., 2001; Arweiler et al., 2004; Auschill et al., 2004, 2005; Al-Ahmad et al., 2007; Dong et al., 2010; Gu et al., 2012; García-Caballero et al., 2013; Prada-López et al., 2015a,b; Quintas et al., 2015b). This is due to the variability in the plaque formation of the different volunteers (Zaura-Arite et al., 2001) or, in some cases, the way the thickness is measured by the authors (Prada-López et al., 2015b). In the present study, the 48 h-biofilm had a thickness of 20–22 μm, which agrees with those found in studies on the PL-biofilm formed *in situ* (21–27 μm; Dong et al., 2010; García-Caballero et al., 2013; Prada-López et al., 2015b; Quintas et al., 2015b). After a single mouthwash, no reduction in the PL-biofilm thickness was found with any of the EO solutions. This finding is consistent with the previous literature regarding EO (Dong et al., 2010; Quintas et al., 2015b); it was only in the case of the 0.2% chlorhexidine solution that some slight reductions could be detected (García-Caballero et al., 2013).

### Influence of a single mouthwash of T-EO and Af-EO on the bacterial viability of the PL-biofilm

In the present series, both EO solutions achieved excellent antibacterial activity, with BV reductions between 57 and 67% and reaching levels of around 5% at 30 s. After the high immediate antibacterial effect that both formulations had, the BV started its slow recovery, albeit more gradually in the case of the Af-EO solution. In any case, both EO mouthwashes were able to maintain the BV under basal levels until 7 h after a single application, maintaining at that moment a 45–50% difference in BV compared to the baseline values.

There are no results in relation to the immediate effect or substantivity for the Af-EO rinse, but some research has been conducted in this field on T-EO solutions. In a previous study of our research group, in which we compared the antibacterial activity of T-EO with those of 0.2% chlorhexidine, we detected that the T-EO antiseptic had an even greater antibacterial effect than the chlorhexidine (Quintas et al., 2015b). In fact, the T-EO rinse had an immediate BV near to zero (it was around 1%) compared to 5% for the 0.2% chlorhexidine. In that study, the T-EO rinse also maintained the BV under baseline levels up to 7 h post-rinsing (Quintas et al., 2015b). In contrast, other studies found that T-EO solutions were not as effective as described in this paper. In this sense, Gosau et al. (2010) found that the BV after a single application of T-EO was around 20%. However, a methodological comment should be made at this point. Gosau et al. used a similar *in vivo* model to the one used in our paper, but the two studies differ in how the T-EO rinses were applied (Gosau et al., 2010). In our experiment, the T-EO were applied as an active mouthwash by the volunteers; in Gosau et al.'s study the disks were removed from the oral cavity and immersed passively in a T-EO solution. This procedure has been found to not be as effective as an active mouthwash at reducing the BV of the PL-biofilm formed *in situ*, particularly in its deepest regions (Prada-López et al., 2015a). Furthermore, differences have been found between not only T-EO rinses but also 0.2% chlorhexidine solutions (Prada-López et al., 2015a). For these reasons, the application methodology of an oral antiseptic should be taken into account when considering the *in situ* antibacterial activity, since oral hydrodynamic forces play an important role in the activation and penetration of the antiseptic in the complex bacterial network that an oral biofilm constitutes (Prada-López et al., 2015a).

The PL-biofilm obtained after 2 days followed a previously described pattern (Dong et al., 2010; Prada-López et al., 2015b; Quintas et al., 2015b), with a BV that was significantly lower in the deeper than the outer layers. This pattern was also followed after the different mouthwashes were applied, except in the immediate samples. Thereafter, the BV gradually increased mainly in the outer layers of the biofilm. These results are also consistent with previous research in the field (García-Caballero et al., 2013; Quintas et al., 2015b).

### Influence of a 4-day protocol of T-EO and Af-EO mouthwashes on the thickness and covering grade of the PL-biofilm

Despite the multitude of studies conducted on a 4-day model of plaque regrowth (Addy et al., 1990; Jenkins et al., 1991; Moran et al., 1997; Rosin et al., 2002; Pizzo et al., 2008, 2013; Ulkur et al., 2013; Prada-López et al., 2015b; Quintas et al., 2015a), very few of them have analyzed the plaque without distortion (Prada-López et al., 2015b; Quintas et al., 2015a). This is not a minor issue, as it has been proved that the three-dimensional structure of the oral biofilm plays a very important role in the biofilm's defense against external agents and is crucial in its development (Wood et al., 2000). In addition, distorting the original structure of the oral biofilm does not permit the measurement of the dental plaque thickness or its BV by layers when it comes to assessing the penetration power of the antiseptic (Prada-López et al., 2015b).

After 4 days of dental plaque accumulation with the performance of daily mouthwashes with sterile water, the obtained thickness (22.8 μm) was consistent with that described in previous publications using the same methodological design (Quintas et al., 2015a; which achieved an oral biofilm thickness of 23.43 μm). In another study carried out by the Arweiler's group (Arweiler et al., 2008) obtained a biofilm of 25.33 μm after immersing the oral biofilm samples in saline twice daily for 5 days. These thicknesses are in line with those obtained by other experiments assessing periods of 3-5 days of evolution (Netuschil et al., 1998; Auschill et al., 2001; Al-Ahmad et al., 2007) when studying the oral biofilm, and varied between 7 and 45 μm.

The two EO mouthwashes were effective at reducing the thickness of the biofilm formed after 4 days compared to the negative control. In fact, the mean thickness measured for the Af-EO solution was almost three times less thick than that obtained with the sterile water (7.9 vs. 22.8 μm); in case of the T-EO solution, the mean thickness was less than half that for the sterile water (9.9 vs. 22.8 μm). These results are in accordance with previous investigations on EO and other antiseptics (Jentsch et al., 2002, 2013; Quintas et al., 2015a). In an earlier study, researchers found a thickness of 10 μm after 4 days of the continuous use of EO (Quintas et al., 2015a). Jentsch et al. (2013), using a scanning electron microscope, obtained a thickness of 10.5 μm after 3 days of the daily use of T-EO. For other antiseptics, the findings vary depending on the mouthwash and the duration of the experiment. For 0.2% chlorhexidine, the results ranged from 6.5 μm after 4 days (Quintas et al., 2015a) to 11.91 μm after 5 days (Arweiler et al., 2008). When a lower concentration was applied (0.12% chlorhexidine), the thickness rose to 14.02 μm after 3 days (Jentsch et al., 2002); in this same study, the antiplaque effect of the stannous fluoride was evaluated, obtaining a thickness of 11.9 μm after the same period of time.

After comparing the thickness obtained for both EO formulations, there was an unexpected result: the Af-EO rinse was more effective than the T-EO rinse at reducing the biofilm's thickness. This finding will be discussed further, along with the CG results.

The CG can be predictive of the adaptation of microorganisms to environmental influences (Al-Ahmad et al., 2008). For this reason, this parameter is crucial and is directly related to the antiplaque effect of an antiseptic agent. Despite this, it has traditionally been forgotten in microbiological studies. In fact, to the best of the author's knowledge, there is only one published article on this issue involving a 4-day PL-biofilm *in situ* (Quintas et al., 2015a). Our results are accordance with this paper, as the CG for the negative control was almost the same (around 73–75%) and it was slightly better for the T-EO rinse (47 vs. 54%). EO solutions were, however, less effective than the 0.2% chlorhexidine, which had a CG of 20% in the same time period. No results were found for the Af-EO rinse, which in the present series had a CG of 33% and were more effective than the T-EO solution at reducing this microscopic parameter.

### Influence of a 4-day protocol of T-EO and Af-EO mouthwashes on the bacterial viability of the PL-biofilm

After 4 days of growth with any disturbing agent other than sterile water, the oral biofilm had a BV of 51%. This observation is consistent with previous research in the field, with results that are slightly over 50% for the BV (Arweiler et al., 2008; Quintas et al., 2015a). In addition, the lowest viability was in the deepest layer, which had BV levels that did not differ too much from those detected after the application of the EO mouthwashes.

This phenomenon has been repeatedly described in the literature, since bacteria located in the lower strata of the biofilm receive fewer nutrients, and so acquire an inactive metabolic state (Netuschil et al., 1998; Pratten et al., 1998). Furthermore, it is deeply related to the greater thickness and density of the biofilm, which makes the correct flow of nutrients and oxygen more difficult to achieve in the deeper layers (Wood et al., 2002).

In the present series, the BV of the 4-day PL-biofilm was reduced by the T-EO and Af-EO solutions (49 and 40%, respectively compared to the negative control). No other studies have been found in relation to the Af-EO, but T-EO rinses have previously been shown to have a greater antiplaque effect in terms of reducing BV of PL-biofilm after 4 days of use, achieving reduction levels close to those of 0.2% chlorhexidine (74 vs. 77%; Quintas et al., 2015a). In another *in vivo* study, Arweiler et al. (2008) found that 0.2% chlorhexidine reduced BV of biofilm by 62% after 5 days of use compared to the negative control.

### Clinical studies on the antiplaque effect of EO formulations

In the present study, a T-EO rinse containing alcohol and an Af-EO solution were used, enabling the effects of one to be compared with those of the other. This is the first study in the literature to compare the antiplaque effect of T-EO and Af-EO solutions in an *in situ* model of non-destructured PL-biofilm grown after 4 days, with the thickness, CG and BV analyzed. However, some studies in the literature have evaluated the effects of both antiseptics at 3–4 days (Marchetti et al., 2011, 2017a,b; Pizzo et al., 2013; Ulkur et al., 2013). In these studies, the authors compare the antiplaque effect assessed by clinical parameters (Marchetti et al., 2011, 2017a,b; Pizzo et al., 2013) or the efficacy of both solutions at reducing *S. mutans* levels (Ulkur et al., 2013). Marchetti et al. (2011) used a 3-day plaque growth model in which the area occupied by plaque was evaluated after performing two daily rinses for 1 min with 20 mL of different EO solutions. T-EO rinses were shown to be more effective at reducing clinical indexes of dental plaque after 3 days (2.18 vs. 2.46). This same group also performed subsequently a similar study (Marchetti et al., 2017a), in which the same Af-EO formulation obtained a similar result (plaque index = 2.45) that was less effective than 0.2% chlorhexidine (plaque index = 1.41). Pizzo et al. (2013) assessed the antiplaque effect of both EO solutions using a plaque index in a 4-day experiment. Equally, they found that the Af-EO rinses were not effective at reducing plaque levels (no differences with regard to the negative control). These macroscopic results do not agree with the microscopic results obtained in the present series, as the thickness and the CG percentage of the PL-biofilm were statistically lower for the Af-EO mouthwash with respect to the T-EO mouthwash. These differences can be explained by the fact that the previous three studies used an alcohol-free formulation (Curasept Daycare, Curaden International AG, Kriens, Switzerland) and another with alcohol (Listerine®, Johnson & Johnson, S. Palomba-Pomezia, Italy) from different manufacturers. In our opinion, this methodological aspect can significantly influence the findings obtained. In fact, recently, another study from Marchetti's group (Marchetti et al., 2017b) compared the alcohol-free and the traditional formulations with ethanol from the same manufacturer (Johnson & Johnson). They found that both solutions were effective compared to the negative control (plaque index = 1.7 vs. 2.3), which confirms the importance of comparing formulations from the same manufacturer. In our case, we used two products from the same manufacturer (Johnson & Johnson, Madrid), which is better for comparison purposes, since the active principles may not be the only ones responsible for the antiplaque effect.

### Antiplaque effect of Af-EO: the importance of the “inactive” ingredients in the formula

In the formulation of oral antiseptics, the active principles play a significant role in the activity against the oral biofilm. However, in the case of chlorhexidine, for example, it has been shown that different formulations with the same concentration of active ingredients produce different results in terms of antimicrobial efficacy (Herrera et al., 2003). The differences in the present series to other findings in the literature may relate to the presence of sodium lauryl sulfate, which is used to dissolve EO (Vlachojannis et al., 2015).

The better microscopic results in the present series of the Af-EO mouthwash in relation to the CG and thickness can be explained by a dual theory. The composition of the two rinses differs, apart from the ethanol, in that sodium lauryl sulfate is present in Listerine® Zero™, but not in Mentol™. This component has been shown to be effective at reducing BV (Jenkins et al., 1991; Petersen et al., 2006; Ledder et al., 2010) and plaque formation (Waaler et al., 1993; Robinson et al., 2006). Its antibacterial effect may be due to the formation of pores in bacterial membranes, which could increase membrane fluidity, reduce phospholipid chains in the membrane, increase the rotation movement of lipid molecules, and change the lateral distribution of proteins and membrane lipids (Petersen et al., 2006). Its effect on dental plaque may be due to a loss of high-density particles present in the cell matrix. Robinson et al. (2006) explained that the removal of structural material by this detergent essentially affects high-density proteins and molecules. This fact could improve the penetrability of the antiseptic, resulting in a greater antiplaque effect. The existence of an inhibitory effect of sodium lauryl sulfate on glucosyl and fructosyl transferases has been identified. These are the enzymes responsible for the synthesis of exopolysaccharides in *S. mutans* (Petersen et al., 2006). This component has been shown to be especially effective against *S. mutans*, as it also reduces lactate formation by 33% (Petersen et al., 2006). In fact, Ulkur et al. (2013) found that both formulations tested in our study had the same effect against these bacteria in a 4-day oral biofilm model. In the present series, the bactericidal effects of the sodium lauryl sulfate may not have manifested, because the Minimum Inhibitory concentration has not been sufficient to affect the BV, although it may have acted on the metabolic level of the bacteria.

As for the other differentiating element, namely ethanol, its effects on the biofilm were studied extensively in the 1990s. Accordingly, the bacteria present in biofilms have adapted physiologically and become more resistant to stress, including that induced by antimicrobial agents (Anwar et al., 1992; Sissons et al., 1996). A possible mechanism by which alcohol resistance above concentrations over 4% could occur is the induction of an adaptive stress response by bacteria (Boutibonnes et al., 1991; Piper et al., 1994; Sissons et al., 1996). In fact, in previous studies, an increase in plaque growth *in vivo* was described in a 4-day model after rinsing twice a day with 50% ethanol (Gjermo et al., 1970). Sissons et al. (1996) reported that alcohol concentrations between 20 and 30% initially produced a rapid inactivation of the bacteria present in the biofilm, but quickly lost its activity and a large resistant population remained unchanged.

In the same sense, long-term studies (Gordon et al., 1985) appear in the literature comparing the use of EO containing ethanol to a negative control of water and its own dissolution vehicle (alcohol at 26.9% and the rest of the excipients). While EO had a significant antiplaque effect after 9 months of continuous use, the dissolution vehicle produced a 7.3% increase in plaque levels after this period of application. These results reinforce the theory that the ethanol *per se* could cause an increase in plaque formation.

## Conclusions

In a 2-day *in situ* oral biofilm model, after a single mouthwash, both essential oils formulations had very high immediate antibacterial activity and a substantivity which lasted for at least 7 h after application. In a 4-day *in situ* oral biofilm model, both essential oils formulations demonstrated a very good antiplaque effect. Although, both essential oils solutions performed well at reducing bacterial viability, the alcohol-free formula performed better at reducing the biofilm thickness and covering grade. Consequently, the alcohol-free essential oils solution represents a reliable option as antibacterial and antiplaque agent for the control of oral biofilm.

## Author contributions

Conception and design the experiments: IT, VQ, IP-L, and DS-Q. Performed the experiments: VQ and IP-L. Analyzed data: VQ, IT, MC, and CB-C. Interpretation of the data: VQ, IP-L. Drafting and revising the manuscript: VQ, IP-L, and IT. Final approval: VQ, IT, and DS-Q. Agreement: VQ, IP-L, CB-C, MC, DS-Q, and IT.

### Conflict of interest statement

The authors declare that the research was conducted in the absence of any commercial or financial relationships that could be construed as a potential conflict of interest.

## Footnotes

^1^Listerine Cool Mint Composition. Available online at: https://www.listerine.com/mouthwash/antiseptic/listerine-cool-mint-mouthwash (Accessed on September 14, 2017). Listerine Cool Mint Zero alcohol composition. Available online at: https://www.listerine.com/products/listerine-zero/listerine-cool-mint-zero-alcohol#product-overview (Accessed on November 1, 2017).

^2^Available online at: www.randomization.com (Accessed on September 03, 2017).

^3^Live/Dead® BacLight™ User Manual. Available online at: https://tools.thermofisher.com/content/sfs/manuals/mp07007.pdf (Accessed on September 30, 2017).
