# Supplementary material for: In Situ Antibacterial Activity of Essential Oils with and without Alcohol on Oral Biofilm: A Randomized Clinical Trial
Source: Front Microbiol. 2017 Nov 23;8:2162. doi: 10.3389/fmicb.2017.02162 (PMC5703870; doi:10.3389/fmicb.2017.02162)
Supplement: Supplementary file 2 [file Table2.DOC]

**
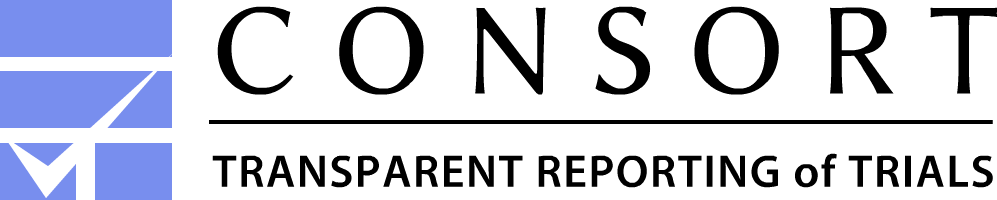
**

**CONSORT 2010 Flow Diagram**

**Enrollment**

**Follow-Up**

**Analysis**

**Allocation**

Assessed for eligibility (n= 30)

Excluded (n= 11)

♦  Not meeting inclusion criteria (n= 11)

♦  Declined to participate (n= 0)

♦  Other reasons (n= 0)

Analysed (n= 6)
♦ Excluded from analysis (n= 0)

Lost to follow-up (n= 0)

Discontinued intervention (loss of a splint) (n= 1)

Allocated to intervention (n= 7)

♦ Received allocated intervention (n= 7)

♦ Did not receive allocated intervention (n= 0)

Lost to follow-up (n= 0)

Discontinued intervention (n= 0)

Allocated to intervention (n= 6)

♦ Received allocated intervention (n= 6)

♦ Did not receive allocated intervention (n= 0)

Analysed (n= 6)
♦ Excluded from analysis (n= 0)

Randomized (n= 19)

Analysed (n= 6)
♦ Excluded from analysis (n= 0)

Lost to follow-up (n= 0)

Discontinued intervention (n= 0)

Allocated to intervention (n= 6)

♦ Received allocated intervention (n= 6)

♦ Did not receive allocated intervention (n= 0)
